# Study protocol: Cerebral characterization of sensory gating in disconnected dreaming states during propofol anesthesia using fMRI

**DOI:** 10.3389/fnins.2024.1306344

**Published:** 2024-02-13

**Authors:** Benedetta Cecconi, Javier Montupil, Sepehr Mortaheb, Rajanikant Panda, Robert D. Sanders, Christophe Phillips, Naji Alnagger, Emma Remacle, Aline Defresne, Melanie Boly, Mohamed Ali Bahri, Laurent Lamalle, Steven Laureys, Olivia Gosseries, Vincent Bonhomme, Jitka Annen

**Affiliations:** ^1^Coma Science Group, GIGA-Consciousness, GIGA Institute, University of Liège, Liège, Belgium; ^2^Centre du Cerveau^2^, University Hospital of Liège, Liège, Belgium; ^3^Anesthesia and Perioperative Neuroscience Laboratory, GIGA-Consciousness, GIGA Institute, University of Liège, Liège, Belgium; ^4^University Department of Anesthesia and Intensive Care Medicine, Centre Hospitalier Régional de la Citadelle (CHR Citadelle), Liège, Belgium; ^5^Physiology of Cognition Research Lab, GIGA-Consciousness, GIGA Institute, University of Liège, Liege, Belgium; ^6^Central Clinical School, Sydney Medical School & NHMRC Clinical Trials Centre, University of Sydney, Camperdown, NSW, Australia; ^7^Department of Anaesthetics & Institute of Academic Surgery, Royal Prince Alfred Hospital, Camperdown, NSW, Australia; ^8^GIGA-CRC—In vivo Imaging—Neuroimaging, Data Acquisition and Processing, GIGA Institute, University of Liège, Liège, Belgium; ^9^Department of Anesthesia and Intensive Care Medicine, Liège University Hospital, Liège, Belgium; ^10^Department of Psychiatry, Wisconsin Institute for Sleep and Consciousness, University of Wisconsin, Madison, WI, United States; ^11^GIGA-CRC—In vivo Imaging—Aging & Memory, GIGA Institute, University of Liège, Liège, Belgium; ^12^Cervo Brain Research Centre, University Institute in Mental Health of Quebec, Québec, QC, Canada; ^13^Consciousness Science Institute, Hangzhou Normal University, Hangzhou, China; ^14^Department of Data Analysis, University of Ghent, Ghent, Belgium

**Keywords:** disconnected consciousness, serial awakening paradigm, propofol sedation, slow wave activity, sensory gating

## Abstract

**Background:**

Disconnected consciousness describes a state in which subjective experience (i.e., consciousness) becomes isolated from the external world. It appears frequently during sleep or sedation, when subjective experiences remain vivid but are unaffected by external stimuli. Traditional methods of differentiating connected and disconnected consciousness, such as relying on behavioral responsiveness or on post-anesthesia reports, have demonstrated limited accuracy: unresponsiveness has been shown to not necessarily equate to unconsciousness and amnesic effects of anesthesia and sleep can impair explicit recollection of events occurred during sleep/sedation. Due to these methodological challenges, our understanding of the neural mechanisms underlying sensory disconnection remains limited.

**Methods:**

To overcome these methodological challenges, we employ a distinctive strategy by combining a serial awakening paradigm with auditory stimulation during mild propofol sedation. While under sedation, participants are systematically exposed to auditory stimuli and questioned about their subjective experience (to assess consciousness) and their awareness of the sounds (to evaluate connectedness/disconnectedness from the environment). The data collected through interviews are used to categorize participants into connected and disconnected consciousness states. This method circumvents the requirement for responsiveness in assessing consciousness and mitigates amnesic effects of anesthesia as participants are questioned while still under sedation. Functional MRI data are concurrently collected to investigate cerebral activity patterns during connected and disconnected states, to elucidate sensory disconnection neural gating mechanisms. We examine whether this gating mechanism resides at the thalamic level or results from disruptions in information propagation to higher cortices. Furthermore, we explore the potential role of slow-wave activity (SWA) in inducing disconnected consciousness by quantifying high-frequency BOLD oscillations, a known correlate of slow-wave activity.

**Discussion:**

This study represents a notable advancement in the investigation of sensory disconnection. The serial awakening paradigm effectively mitigates amnesic effects by collecting reports immediately after regaining responsiveness, while still under sedation. Ultimately, this research holds the potential to understand how sensory gating is achieved at the neural level. These biomarkers might be relevant for the development of sensitive anesthesia monitoring to avoid intraoperative connected consciousness and for the assessment of patients suffering from pathologically reduced consciousness.

**Clinical trial registration:**

European Union Drug Regulating Authorities Clinical Trials Database (EudraCT), identifier 2020-003524-17.

## 1 Introduction

**Figure 1:**
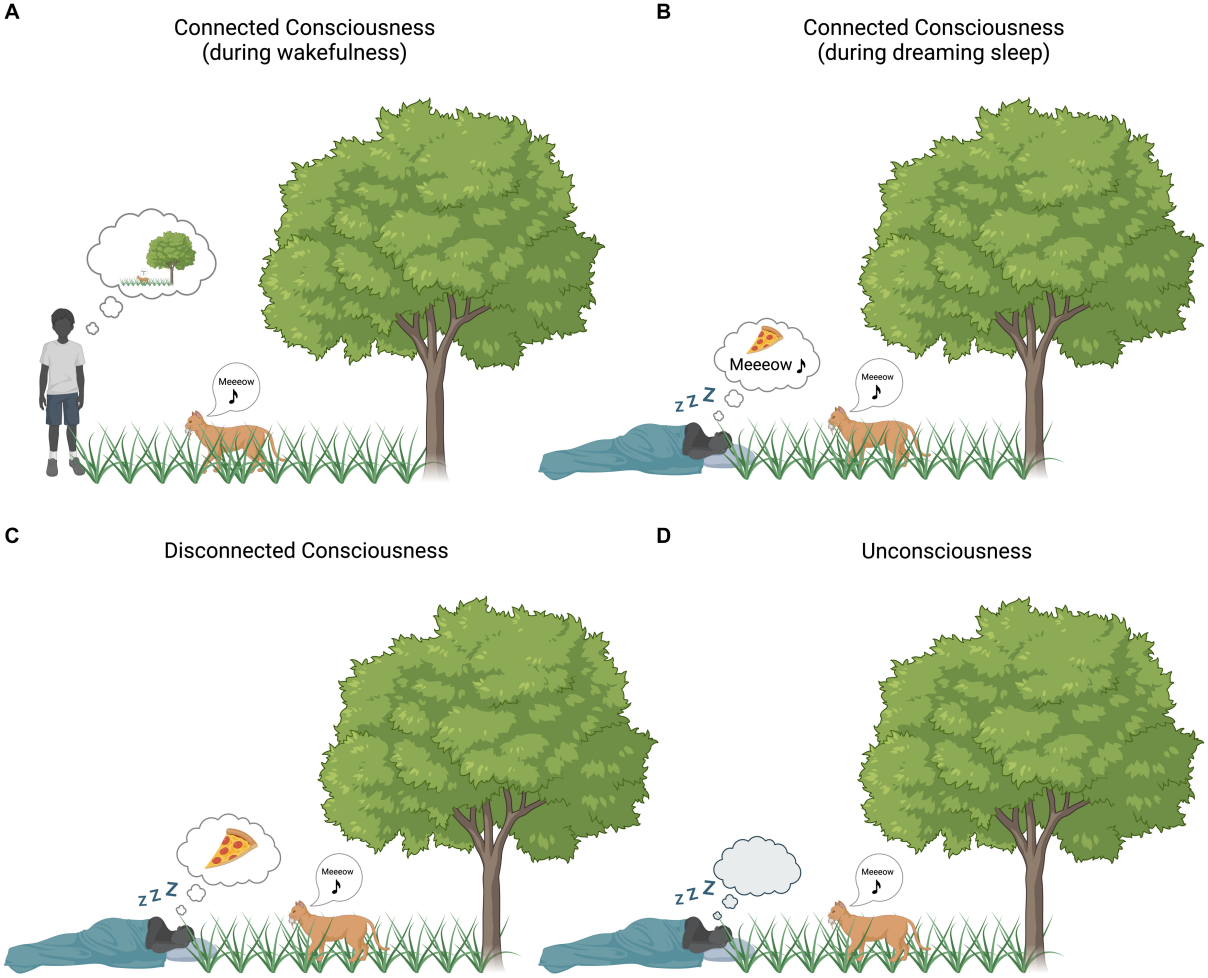
Environmental (dis)connection and consciousness. Bubble clouds represent subjective experience [i.e., (un)consciousness]. **(A)** Connected consciousness during wakefulness: the boy’s subjective experience is strongly influenced by the surrounding environment. **(B)** Connected consciousness during dreaming: the subjective experience of the sleeping boy is partially influenced by the surrounding environment (the boy is dreaming of eating pizza) and, at the same time, sounds from the surrounding environment (in this case the cat’s sound) are incorporated into his experience. **(C)** Disconnected consciousness during dreaming: the boy’s subjective experience is not influenced by the surrounding environment (the boy is only dreaming about eating pizza, no environmental stimuli are incorporated into the ongoing experience). **(D)** Unconsciousness: the boy is devoid of any experience, whether originating internally or externally. Created with BioRender.com.

During wakefulness, under normal conditions, our subjective experience (i.e., consciousness) is usually strongly influenced by external, environmental stimuli, that is, our consciousness is connected to the physical world [i.e., connected consciousness (CC)]. When transitioning to dream states (such as physiological dreaming or anesthesia-induced dreaming), our subjective experience often continues to be remarkably rich. Highly vivid sensory experiences during dreaming are frequently reported, yet they are usually unaffected by external stimuli, i.e., our consciousness is disconnected from the physical world [i.e., disconnected consciousness (DC)] (Figure 1). Corticocortical connections are functionally preserved to generate dreaming experiences, yet, somewhere in the thalamocortical stream, stimuli from the external world are blocked from conscious processing. Currently, a major obstacle to identify the cerebral gating mechanisms underlying sensory disconnection is the lack of behavioral differentiation between disconnected and connected conscious states in the neuroimaging literature. Previous research conducted on anesthetized participants has usually assumed a binary context, comparing brain activity acquired during wakefulness with brain activity acquired during presumed unconsciousness, where unconsciousness was inferred from participants’ unresponsiveness. Inferring the presence or absence of consciousness from (un)responsiveness^1^ has now been widely shown to be inaccurate, as unresponsiveness does not always correspond to unconsciousness (Sanders et al., 2012). Studies using the isolated forearm technique (i.e., assessing responsiveness by preventing muscle relaxants to act on one of the forearms) revealed in fact that up to 37% (Sanders et al., 2012; Linassi et al., 2018) of anesthetized patients, despite looking deeply asleep, were conscious of external stimuli (Sanders et al., 2012; Linassi et al., 2018). However, more recent estimates from clinical practice suggest that 5–10% of patients in routine clinical care experience these episodes (Sanders et al., 2017; Lennertz et al., 2023). Episodes of intraoperative dreaming are more frequent and have been estimated to occur in 22–59% of anesthetized, unresponsive patients (Leslie et al., 2007; Errando et al., 2008; Noreika et al., 2011). This implies that the supposed neural signature of consciousness gathered from classical anesthesia studies might conflate disconnected, connected consciousness, unconsciousness or the alternation between these states. We here propose that sensory perception of external stimuli can fluctuate while consciousness remains constant and independently of arousal (e.g., during dreaming), resulting in disconnected and connected dream-like experiences.

To disentangle between these different states and investigate the neural basis of (dis)connected consciousness,^2^ more recent studies have resorted to serial awakening paradigms during sedation (Radek et al., 2018; Scheinin et al., 2021; Casey et al., 2022a; Valli et al., 2023). With serial awakenings, participants are directly questioned about their mental activity that was ongoing before being awakened, minimizing the lack of explicit recall which is common due to amnesic effects of anesthesia or sleep (Siclari et al., 2013, 2017). However, contrasting episodes of (unresponsive) disconnected consciousness with other behavioral states [e.g., responsive wakefulness (Scheinin et al., 2021; Valli et al., 2023), self-reported wakefulness (Casey et al., 2022a)] impedes the segregation of the neural correlate of disconnected consciousness. Indeed, instead of reflecting the neural correlates of sensory (dis)connection, such contrasts could reflect differences in responsiveness or arousal system. A common assumption, also shared in these studies, is that connected consciousness is wakefulness, but this is not necessarily always the case, not least due to the complexity of defining wakefulness. There is evidence that during anesthesia patients often incorporate auditory and somatosensory stimuli into their dreams or perceive such stimuli in a dream-like state (Leslie et al., 2007, 2009; Leslie, 2010; Noreika et al., 2011; Radek et al., 2018). These episodes have been referred to as “near-miss awareness” (Leslie, 2010). Evidence that environmental stimuli can be perceived during dreaming without necessarily triggering wakefulness is also documented in sleep studies (Nielsen, 1993; Leslie and Ogilvie, 1996; Schredl et al., 2009). As the search for the neural correlates of consciousness (NCC) has been refined over the years by distilling the proper NCC from its prerequisites and consequences, the same should be attempted in the search for the NC of (dis)connected consciousness.

Although there is suggestive evidence indicating that a breakdown in cortical effective connectivity (Massimini et al., 2005) might underlie the loss of consciousness in anesthesia (Boly et al., 2012) and sleep (Esser et al., 2009), the challenge heightens when attempting to pinpoint the specific mechanism responsible for the loss of environmental connection during these states. While we know that sensory stimuli reach primary sensory regions during presumed unconscious and dream states, it is unknown how they are processed in disconnected states in both primary and secondary regions. In REM sleep (Funk et al., 2016) and mild sedation with propofol (Boly et al., 2012), deactivation of primary areas has been shown to coexist with the activation of secondary/associative regions, favoring top-down over bottom-up cortical signaling. This imbalance between top-down/bottom-up information flow may be one of the potential mechanism for the decoupling of consciousness from environmental connection (Murphy et al., 2011; Funk et al., 2016; Andrillon and Kouider, 2020; Casey et al., 2022b). Recent studies also suggest a role in disconnectedness for specific thalamic nuclei, depressing cortical function and compromising thalamocortical information flow (Liu et al., 2015; Feng et al., 2017). Finally, a mechanistic role for local slow wave activity (SWA)–δ band (1–4 Hz) frequency oscillations—has been proposed for inducing disconnectedness. Local SWA has been found in all those states that present episodic coupling of conscious experience with disconnection from the environment: SWA was recorded during REM sleep at the level of primary sensory and motor cortices (Funk et al., 2016) and has long been found in NREM sleep and anesthesia (Murphy et al., 2011). In propofol anesthesia, SWA saturation has been reported to deactivate the thalamus and primary cortices, interrupting wake-like brain activity to external stimuli, thus probably inducing a state of disconnectedness (Mhuircheartaigh et al., 2013).

Here, we propose to identify unresponsive connected and disconnected dream-like states by delivering auditory stimuli during propofol-induced mild sedation and serially awaking healthy participants. We will collect subjective reports about mental activity prior to awakening (assessing dreaming/consciousness) and stimulus perception (assessing connectedness), while ensuring unresponsiveness throughout the experiment and minimizing the risk of arousals. The cerebral activity of participants will be recorded by means of functional MRI. During the auditory stimulation session, we will play series of sounds following the oddball rule, in which trains of beeps of the same frequency (i.e., standard sounds) are occasionally interrupted by a beep of a different frequency (i.e., the deviant or “oddball” sound). This way, we will be able to investigate not only the difference in the perception of sounds during connected and disconnected consciousness, but also whether standard and deviant sounds are processed differently in the two conditions.

Capitalizing on the enhanced spatial resolution of BOLD fMRI, we will (1) characterize stimulus processing within several thalamic nuclei, and primary and secondary cortices during connected (CC) and disconnected consciousness (DC). To this end, we will conduct a hypothesis-driven ROI analysis, in which we will test various hypotheses on the involvement of thalamic nuclei (*Hypothesis 1*), primary auditory cortex (*Hypothesis 2*) and secondary areas (*Hypothesis 3*) in the processing of auditory stimuli during CC and DC states. The ROI analysis will be complemented by an exploratory whole-brain analysis aimed at identifying other regions potentially involved in sensory disconnection. If *Hypothesis 1–2* prove true, it would indicate that already at a basic level of stimulus processing there is a difference between CC and DC. To investigate this further, we aim (2) to characterize changes in functional connectivity between the thalamus and primary auditory cortices with the rest of the brain in CC and DC. We hypothesize (*Hypothesis 4*) that the functional connectivity between the thalamus [e.g., the pulvinar (Kanai et al., 2015; Sanders et al., 2021)] and primary auditory cortices will be stronger in CC compared to DC. If this hypothesis is confirmed (together with Hypothesis 1–2) it would suggest that some gating mechanism already occurs at the thalamic level. However, this difference in brain activity might be necessary but not sufficient to cause sensory disconnection: that is, a weakened connection between the thalamus and primary auditory cortices does not imply that sensory stimuli are *entirely* blocked from cortical processing via the gating action of the thalamus [thalamic gate hypothesis (Andrillon and Kouider, 2020)]. It would however indicate that differential processing of stimuli in CC and DC already occurs at the thalamic level. This thalamic modulation of sensory inputs could in turn affect cortical processing, leading to a cortical gate, that is, loss of information propagation to higher cortices due to a disruption in functional connectivity (cortical gating hypothesis). In this respect, we hypothesize (*Hypothesis 5*) that the functional connectivity between primary and secondary auditory cortices will be stronger in CC compared to DC. Voxel-to-voxel functional connectivity analysis will be conducted for standard and deviant sounds separately: we hypothesize (*Hypothesis 6*) that different processing for deviant and standard sounds will be present in CC but absent in DC. Finally, we aim (3) to quantify high-frequency BOLD oscillations, which have been shown to track sleep slow waves (Song et al., 2022), in selected thalamic nuclei and in primary and secondary cortices. We hypothesize (*Hypothesis 7*) that high-frequency BOLD oscillations will be lower in high-order/first-order thalamic nuclei and in primary and secondary sensory cortices during CC compared to DC.

In summary, we propose an fMRI experiment that systematically differentiates connected and disconnected conscious states by delivering auditory stimuli and serially awakening participants sedated with propofol to assess the conscious state and stimulus perception through subjective reports. Through activation and connectivity analyses of collected fMRI data, we will investigate whether sensory disconnection is caused by altered activity at the level of thalamus, primary regions, or, higher up, due to a lack of stimulus integration in associative areas. This project will provide fundamental insights on the neural correlates of sensory disconnection.

## 2 Methods

### 2.1 Inclusion and exclusion criteria

Participants will be screened through an online form, an in-person interview and a medical examination. The initial phase of screening using the online form will select healthy, right-handed, non-smoking, MRI-compatible subjects without psychiatric and neurological disorders, propensity for nausea, recurrent nightmares, memory and hearing impairments, substance abuse, cannabis use in the three months preceding the study and regular alcohol consumption (i.e., everyday). During the in-person interview it will be verified that the above inclusion/exclusion criteria are fulfilled to avoid oversights or errors in filling out the form. Furthermore, we will select participants who have a low risk of obstructive sleep apnea through the StopBang questionnaire (Low Risk: Yes to 0–2 questions) (Chung et al., 2008, 2012) and low levels of anxiety through the scales “Novelty Seeking” and “Harm Avoidance” of the Temperament and Character Inventory self-rating questionnaire (Cloninger et al., 1993). We will only include participants with average or above average scores on the “Novelty Seeking” scale (i.e., ≥16.5 for men and ≥ 16.3 for women) and participants with average or below average scores on the “Harm Avoidance” scale (i.e., ≥14.5 for men and ≥ 17.5 for women) (Pélissolo and Lépine, 2000). We control for obstructive sleep apnea because of the known respiratory depression effects of propofol. We take into account the predisposition to anxiety as anxious participants might require higher propofol concentrations to achieve loss of responsiveness compared to non-anxious participants, impairing subsequent recovery of responsiveness and report collection. Participants who meet these criteria will be visited by an anesthesiologist with an evaluation similar to a pre-surgical examination, including a physical examination, full review of the patient’s medical, surgical and allergological history, treatment and intubation score and any potential contraindication to propofol sedation. Finally, alcohol consumption will be forbidden for 48 h preceding the experiment. A proper sleep hygiene is encouraged in the 2 to 3 days prior to the study. Participants are required to refrain from drinking and eating six hours before the start of the experiment.

### 2.2 Experimental setup

**Figure 2:**
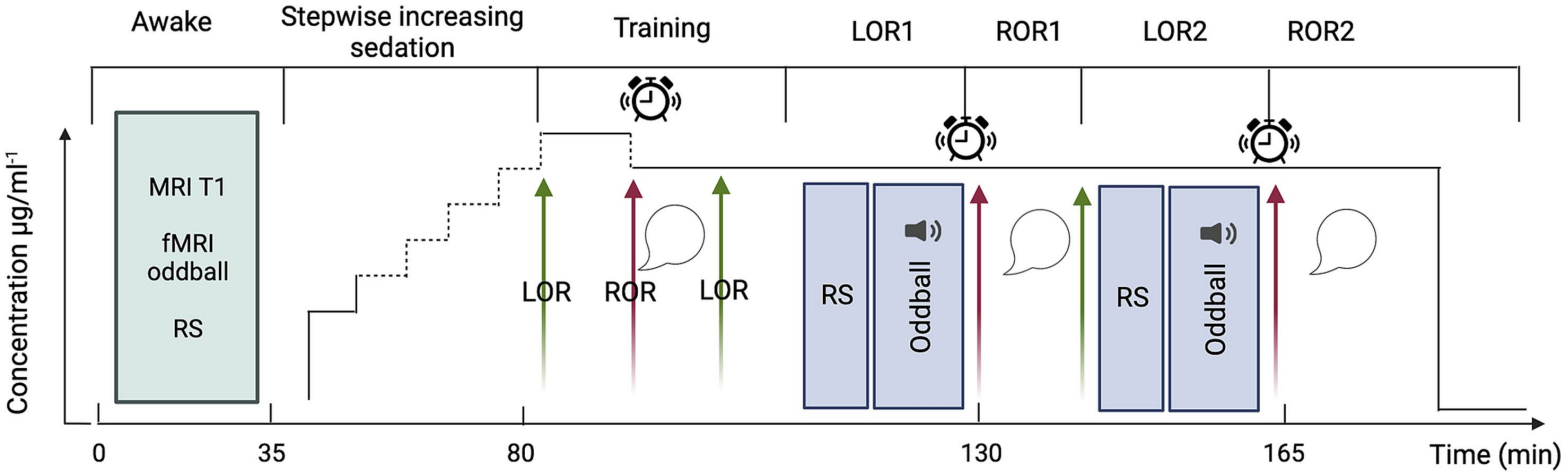
Schematic representation of the experimental design. Green arrows indicate loss of responsiveness; red arrows, regain of responsiveness; sound symbol, auditory oddball stimulation; alarm clock, awakening attempt; speech bubble, collection of subjective reports. fMRI acquisitions will be conducted throughout LOR1 and LOR2 and concluded prior to the initiation of the awakening attempt. Created with BioRender.com.

In short, the present experiment will comprise four main phases (schematic representation in Figure 2): (1) acquisition of MRI data in awake participants during rest and auditory stimulation, (2) gradual sedation with propofol (~45 min), (3) training session of sedated participants for ~20 min (see below for an explanation) and (4) acquisition of MRI data in sedated participants during rest and auditory stimulation, both repeated twice. During the awake phase a structural (T1) image will be collected prior to the acquisition of functional scans during rest (~10 min) and auditory stimulation (~15 min; for a detailed description of the auditory paradigm, see the section “fMRI experimental design and auditory paradigm”). To prevent the comparison of connected and disconnected consciousness from being contaminated by correlates of (un)responsiveness we will ensure that participants were unresponsive prior to the start of the experimental sessions and awakening. We will also assess their state of wakefulness before and after the experimental sessions to minimise the risk of arousals and confounding correlates of wakefulness with those of (dis)connected consciousness.

From the beginning of the sedation, to monitor responsiveness, participants will be instructed to perform a continuous task of alternately pressing the left and right keys of a box-shaped keypad. Propofol will be infused until loss of volitional motor activity which will be used as a proxy for loss of responsiveness (LOR). LOR will be defined as three consecutive minutes in which the participant has stopped pressing keys and neither speaks nor moves spontaneously. Propofol will be administered by a computer-controlled continuous infusion (target-controlled infusion—TCI) using a pharmacokinetic model to achieve stable plasma and effect-site propofol concentration (Schnider et al., 1999). The initial target for induction will be set at 1 μg.ml^−1^, and progressively increased (waiting five minutes between each increase in concentration) until LOR as follows: from the initial target, propofol concentration will be increased by a step of 0.5 μg.ml^−1^ to 1.5 μg.ml^−1^; if LOR will not be reached at 1.5 μg.ml^−1^, propofol will be increased by a step of 0.2 μg.ml^−1^ until 1.9 μg.ml^−1^. If LOR will not be reached at 1.9 μg.ml^−1^ propofol will be increased by steps of 0.1 μg.ml^−1^ until LOR. This will ensure to determine, for each subject, the precise concentration at which LOR occurs. This approach minimizes the risk to exceed the dose required to reach LOR. The higher the dose, the more difficult the recovery of responsiveness may become. Since the aim is to target LOR and not loss of consciousness, the maximum propofol concentration will be set at 4 μg.ml^−1^. This sedative dosage will also allow the participants to remain in spontaneous ventilation. When participants lose responsiveness, we will wait 5 min for the drug to stabilize, and then we will begin the training session. The goal of the training session is to fine-tune the propofol concentration to maximize the chances of having both LOR and intelligible reports upon regain of responsiveness (ROR) at the same propofol concentration. During the training session (outside the MRI scanner bore, but on the MRI table), we will attempt to awaken participants by performing an arousal protocol that will consist of (1) calling the volunteer aloud through the MRI microphone for up to 2 times, (2) if unsuccessful, lightly shaking volunteer’s shoulders for up to 2 times, (3) if unsuccessful, applying moderate painful stimulation, i.e., pinching the skin of the forearm for up to 2 times. If, after three runs of the protocol, participants are still not responsive, we will decrease the propofol concentration by 0.1 μg.ml^−1^, wait five minutes and repeat the arousal protocol a second time. This process is repeated until participants are able to recover responsiveness. Once responsiveness has been recovered, participants will be asked questions (see “interviews following ROR”) to verify their state of (dis)connectedness and (un)consciousness. If participants are unable to speak intelligibly, the concentration of propofol will be reduced by steps of 0.1 μg.ml^−1^ (always waiting 5 min between steps) until reports become intelligible. Once the right concentration has been identified, we will wait for participants to spontaneously lose responsiveness again, and we will end the training session with participants unresponsive (i.e., LOR1).

LOR1 will mark the beginning of the experimental session. After LOR1, we will acquire 10 min of resting-state (RS) fMRI followed by 15 min of task-based fMRI, i.e., passive listening to a sequence of oddball auditory stimuli. After concluding the fMRI acquisition and without altering the drug concentration, we will attempt to awake participants by performing the arousal protocol described above. If the participant does not regain responsiveness after one execution, the protocol will be repeated a maximum of two times. If the participant does not regain responsiveness after three executions, the participant will be defined unarousable. In case of ROR, participants will be questioned about their experience during the period of unresponsiveness to determine whether they were (un)conscious and (dis)connected (see “interviews following ROR”). After the first ROR (ROR1), we will wait a maximum of 10 min for the participant to spontaneously lose responsiveness a second time (LOR2). If the participant does not lose responsiveness in the 10 min following ROR1, we will increase the propofol concentration up to three times in 0.1 μg.ml^−1^ increments. If, after increasing the concentration, the participant does not reach LOR2, the LOR attempt will be considered unsuccessful, and the experiment will be terminated. In the case that LOR2 succeeds, the procedure described above for ROR1 will be repeated for ROR2. Finally, the end of the experiment is marked by termination of the drug infusion. This protocol has been fine-tuned based on previous work of our team (Bonhomme et al., 2016).

### 2.3 Interviews following ROR

After every successful ROR, participants will be subjected to the following 6-question interview (and provided with the following possible answers):

1. Did you have any sensations or thoughts before you were awakened? Yes/No/Not sure/Nothing
2. Did you hear the tones? Yes/No/Not sure
3. Do you think you were awake, having a dream or unconscious? Awake/Dream/Unconscious/Not sure
4. Did you hear one or two different tones? One/Two/Not sure/Nothing
5. Was this experience more centered on yourself or on the environment? Myself/Environment/Nothing
6. Did you rather think, or did you see many things? Think/See/Not sure/Nothing

The first and third questions will verify that participants were (un)conscious during the period of unresponsiveness and the second question will verify the (dis)connectedness of participants during the period of unresponsiveness. The last three questions will serve two purposes: to gather more detailed information on the experience during the period of unresponsiveness and to check the consistency of the reports. Probing the presence of the experience/perception of sounds will strengthen the reliability of the answers to the first two questions (e.g., the participant might answer that he/she did not hear any sounds, but then answer the question “did you hear one or two tones?” with “two”). This interview will identify four different states: (1) awakening without any recall of experiences; (2) connected dreaming; (3) disconnected dreaming and (4) wakefulness. State 1 will be discarded as it cannot be classified in either of the two categories of interest in this study. Connected consciousness (i.e., connected dreaming) will be considered to have occurred during the unresponsive period if participants answered “Yes” to questions number 1–2 and disconnected consciousness if participants replied “Yes” to question 1 and “No” to question 2. In the case of conflicting answers, we will consider participants to have been connected conscious if they provide a positive response to at least one question amongst numbers 1,5,6 or if they respond with “having a dream” to question number 3, in addition to a positive response to at least one question amongst numbers 2 and 4. Participants responding with “awake” to question number 3 will be excluded, as they will be considered not to be in a state of connected consciousness during a dream-like state but rather awake. If participants replied with “No/Nothing” to question number 2–4 and positively to at least one of the questions investigating their experience (i.e., question number 1,3,5,6) they will be considered having been disconnected conscious during the unresponsive period. Participants will be acquainted in advance with the different questions and possible answers to ensure full understanding of each question. To rule out potential arousals during fMRI acquisitions, subjects will be monitored continuously throughout the acquisition via an eye-tracking camera (EyeLink 1000plus system from SR Research, Ltd) – the eye-tracker will be used for online monitoring but not for offline analysis. In case of eye opening, MRI acquisition will be interrupted, and we will wait for participants to spontaneously fall unresponsive again. If, after 15 min, LOR does not occur, we will increase the propofol concentration up to three times in 0.1 μg.ml^−1^ increments.

### 2.4 fMRI experimental design and auditory paradigm

**Figure 3:**
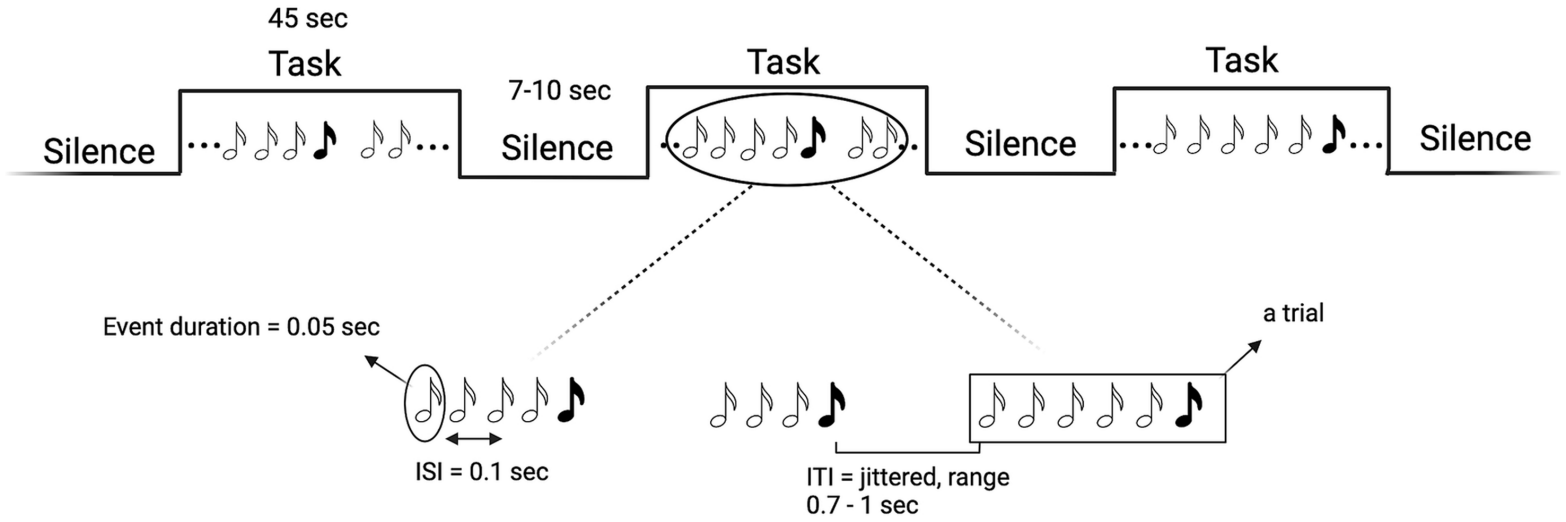
Schematic representation of the mixed block/event-related design (top) and of the “Mixed35” oddball rule (bottom). White musical notes denote standard sounds, black notes deviant sounds. Created with BioRender.com.

We chose a mixed block/event-related design (Figure 3) in which trials of auditory stimuli are interspersed with blocks of silence of varying durations (15 blocks in total, each lasting 45 s and containing 30 trials on average). This design allows for the simultaneous modelling of the transient, trial-related activity, and the sustained, task-related BOLD activity. That is, by alternating silence blocks with task blocks (i.e., blocks with trials with auditory stimuli) we can optimize the sensitivity for discriminating events within trials and all events combined within a block. Blocks with only standard events will be alternated with blocks containing both standard and deviant events. Standard and deviant blocks will alternate in pseudorandomized order (ABBA…), in which no more than two identical types of blocks can follow one another. The length of the silence blocks will be randomized in 1-s steps in intervals of 7–10 s. Based on previous studies (Bekinschtein et al., 2009), each event (both standard and deviant sounds) will lasts 0.05 s; inter-stimulus interval (ISI) will be fixed at 0.1 s and inter-trial interval (ITI) will be jittered in 0.05-s steps between 0.7 to 1 s. We will use a variant of the “classic oddball” paradigm (Figure 3), in which trials will consist of a randomized number of repetitions (i.e., 3–5) of standard events plus one deviant event. Standard sounds will have a frequency of 100 Hz and deviants of 500 Hz. The total length of the auditory stimulation will be 15 min. Each task block will last 45 s (to have both a frequency still below the recommended 128 s high-pass filter, but also a reasonable number of trials). The parameters selected for the auditory stimulation are the result of the efficiency and collinearity analyses we performed to optimize the efficiency of our design. Sounds will be delivered via a Serene Sound Digital MRI-compatible system.

### 2.5 Efficiency and collinearity analyses for optimizing the auditory paradigm to fMRI

**Figure 4:**
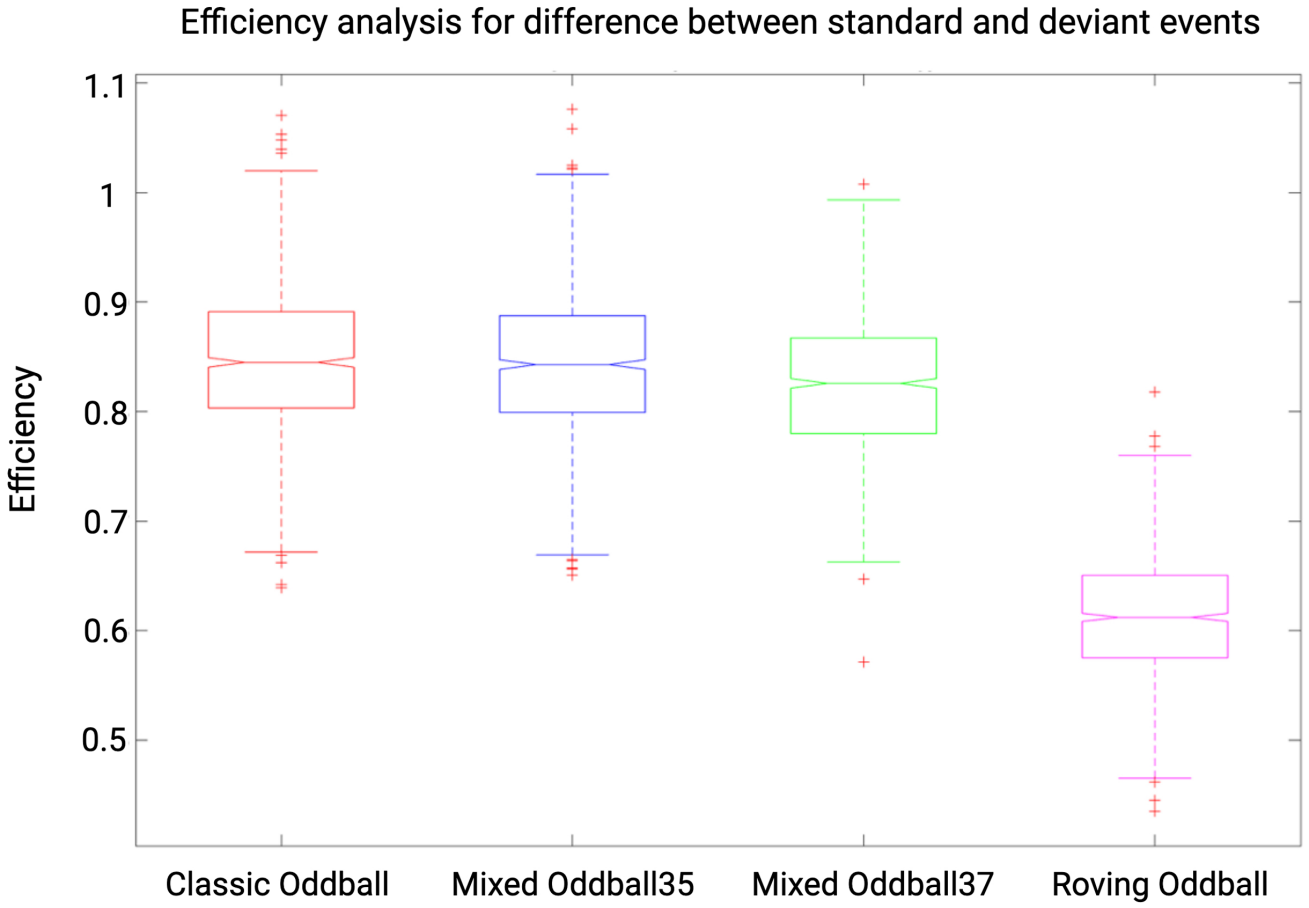
Efficiency results for four auditory paradigms. Results shown are for contrast standard-deviant, TR = 0.842 and for an experiment duration of 900 s (i.e., 1,125 scans). Efficiency values are reported for “Classic oddball,” “Mixed Oddball35,” “Mixed Oddball37,” and “Roving oddball” with silence blocks of duration 7–10 s and in pseudorandomized order (ABBA). Whiskers corresponds to approximately +/−2.7σ and 99.3 percent coverage. We selected the “Mixed Oddball35” for its combination of high efficiency and the reduction of expectation achieved through the randomization of standard sounds.

With the following analyses, we sought to maximize the efficiency of the auditory paradigm which led to the protocol described in the section “fMRI experimental design and auditory paradigm.” Estimation of the efficiency can be defined as “a measure of the reliability with which model parameters are estimated”(Mechelli et al., 2003). The efficiency of a design strongly “affects the sensitivity with which experimental effects are detected” (Mechelli et al., 2003). In order to find the (*a priori*) most efficient design to detect our effects of interest, we manipulated the temporal distribution of events, resulting in several designs whose efficiency was estimated *a priori* and then compared. The variables manipulated were the length of the silence blocks (i.e., randomized in 1-s steps in intervals of 7–20 s, 7–10 s, 15–20 s, 10–15 s), the ordering of standard and deviant blocks (i.e., interleaved order or pseudorandomized order in which no more than two identical types of blocks can follow one another), the type of oddball paradigm. We selected four different types of oddball paradigms for comparing their efficiency, of which only one was chosen for the experiment. In the “classic oddball” paradigm (Squires et al., 1975), trials consist of four standard sounds and one deviant, where the deviant is defined by a change in frequency. The “roving oddball” (Garrido et al., 2007) differs with each trial presenting sounds of the same frequency and starting a new trial with a different frequency, making the first event of a trial a deviant. We also designed two “mixed oddballs,” in which trials follow the “classic oddball” rule but with the difference that the number of repetitions of standard events is randomized, between three and five (“Mixed Oddball35”) or between three and seven repetitions of standards (“Mixed Oddball37”). The efficiency analysis was conducted by comparing all possible combinations of parameters (i.e., ISI, ITI, stimulus and block duration) for the different oddball paradigm designs. Please note that the efficiency calculation is related to the number of scans (i.e., to a given TR and duration of experiment), and specific to a given contrast. We calculated the efficiency for TR = 0.842 s, 900 s duration of the experiment and for the following three contrasts: main effect of the standard response, main effect of the deviant response and the difference between standard and deviant responses. For more information on how we computed efficiency, see our repository on GitHub “Efficiency-Analysis-fMRI-mixed-design,” where each step of the analysis is detailed: https://doi.org/10.5281/zenodo.8117861. In addition to the efficiency analysis, we also performed a collinearity analysis in SPM to estimate the extent to which our two events (standard and deviant) were collinear (i.e., whether their responses correlated with each other)—see our GitHub repository for more details on how to compute collinearity in SPM. The design that was most efficient and with least collinearity was the “Mixed Oddball35” with 7–10 s silence blocks and pseudorandomized order (mean efficiency for the difference between standard and deviant events = 0.843). As depicted in Figure 4, “Mixed Oddball35” was found to have comparable efficiency with the “Classic oddball” (mean efficiency for the difference between standard and deviant events = 0.845). We chose the “Mixed Oddball35” for the experiment as randomizing the number of repetitions of the standard sounds has the advantage of decreasing expectation.

### 2.6 MRI data collection

MRI data will be collected with a 3 T Magnetom Prisma Fit scanner (Siemens, Erlangen, Germany) equipped with a 20-channel array receiver head–neck coil. For rs-fMRI and task based-fMRI, the scanning parameters will be as follows: echo-planar imaging (EPI) with multi-band acceleration factor of 6, 7/8 phase partial Fourier, 2.25 mm slice thickness, no gap between slices, 2.25 mm x 2.25 mm in-plane spatial resolution, 842 ms repetition time (TR), 30 ms echo time (TE), 52° flip angle, 207 mm x 225 mm field of view (FOV) and a matrix size of 92 × 100. For anatomical reference, a high-resolution T1-weighted image will be acquired for each subject during the awake session (T1-weighted 3D magnetization-prepared rapid gradient echo (MPRAGE) sequence, TR = 1900 ms, TE = 2.19 ms, inversion time (TI) = 900 ms, sagittal orientation, 224 slices, 1 mm slice thickness, FoV = 256×240 mm^2^, matrix size = 256x240x224, voxel size = 1x1x1 mm^3^, GRAPPA R = 2 acceleration factor in phase-encoding direction (AP).

### 2.7 Variables of interest, randomization, and blinding

In this experiment, the dependent variable will be the BOLD signal (during RS/task sessions). The independent variables will be both the connected/disconnected condition and the type of sounds delivered, i.e., standard or deviant events or silence. The connected/disconnected condition cannot be randomized, given its unpredictability, i.e., it is impossible to predict which individuals will report being connected and which will report being disconnected. In contrast, several parameters of the auditory paradigm will be randomized (e.g., the number of repetitions of standard events). Similarly, the temporal order of the waking and sedation sessions cannot be randomized given the use of anesthetics: data acquisition after the end of sedation would in fact correspond more to acquiring data during the recovery than during the awakening phase. Finally, the order of the RS and task sessions will not be randomized due to time constraints: since the collection of reports must occur immediately after the task session, if the task preceded the RS session, we would be forced to collect reports after the task and then wait for the participants to lose responsiveness again to acquire the 10 min of rs-fMRI, considerably extending acquisition time. Data preprocessing and analysis will be performed blind to the conditions of the experiment.

### 2.8 Sample size calculation

**Figure 5:**
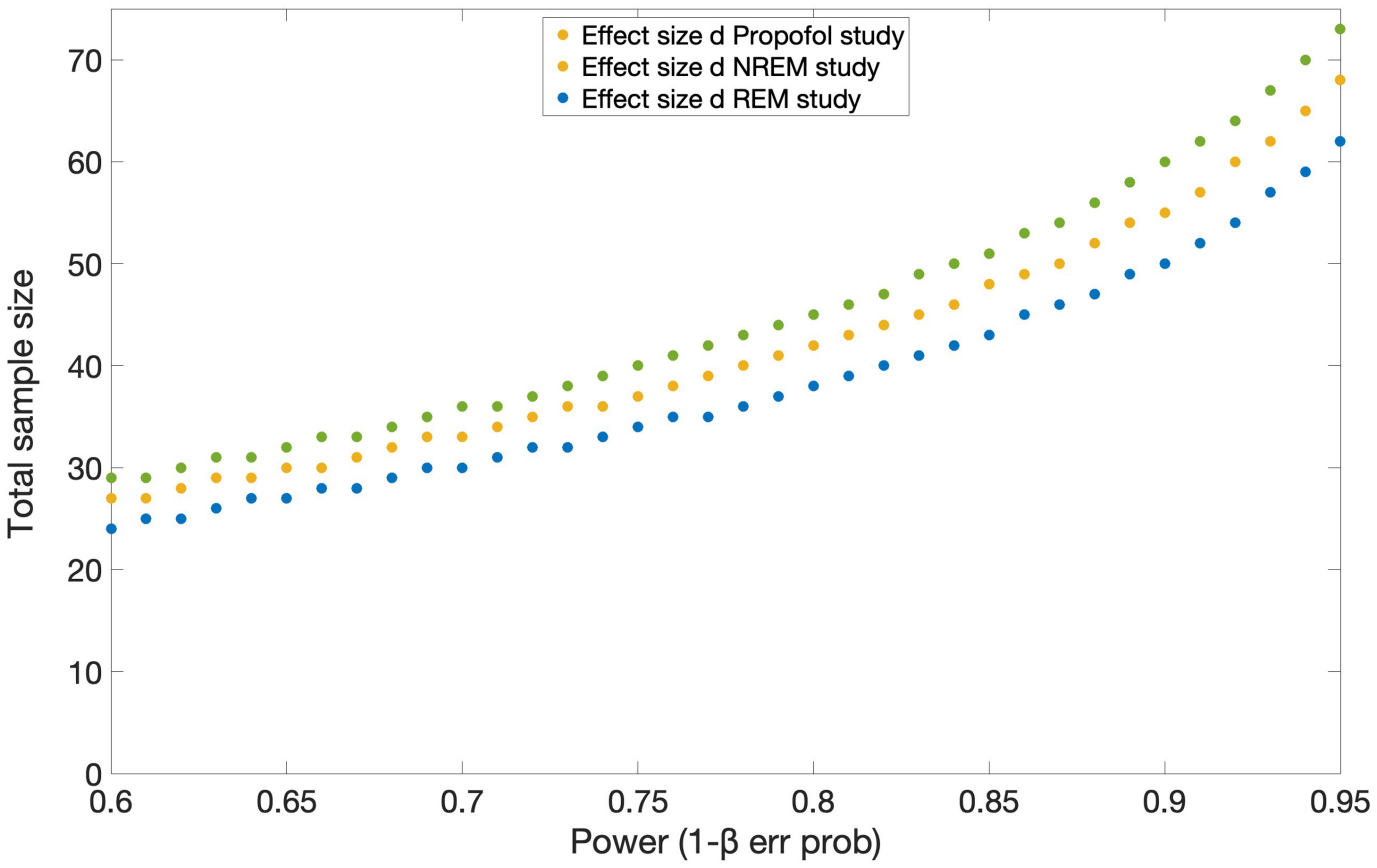
Results of the power analysis based on the effect sizes obtained from three studies investigating the difference between connected and disconnected consciousness using EEG. Estimated total sample sizes for power 0.6–0.95 are shown for each of the effect sizes reported in the studies conducted under propofol sedation [yellow circles; Casey et al. (2022a)], during NREM (blue circles) and REM (green circles) sleep. α err prob. = 0.05.

To the best of our knowledge, no study has investigated cerebral changes between connected and disconnected consciousness using fMRI. Hence, no effect size was available in the literature for a power calculation with a similar setup as the current experiment. However, we were able to make an approximate estimate of the total sample size required based on EEG studies investigating sensory disconnection during propofol anesthesia (Casey et al., 2022a), REM (own data, to be submitted) and NREM sleep (own data, to be submitted). We estimated the effect size with Cohen’s *d* from the means and standard deviations of each group, and we performed power calculations (two tailed t-tests) by setting the desired α at 0.05 and power at 0.95. In the first study conducted under propofol sedation, the effect size of the difference between CC and DC in occipital delta power was 1.0 and the allocation ratio N2/N1 was 2.78. In the REM and NREM sleep studies, the effect size of the difference between CC and DC in event related potentials was 0.85 with allocation ratio N2/N1 = 1 and 0.94 with allocation ratio N2/N1 = 0.71, respectively. The total estimated sample size is of 70 sessions according to power calculations based on the effect size of the propofol study; of 64 sessions based on the effect size of the NREM study and of 74 sessions, based on the REM study’s effect size (see Figure 5). Taking the most conservative estimate based on the smallest effect size of the three studies, and accounting for an 8% dropout (e.g., impossible to reach LOR or ROR), we plan to collect 40 subjects, for a total of 80 sessions (two per subject). This power analysis was conducted in G*Power 3.1.9.7 software (Faul et al., 2007, 2009) and the results plotted in MATLAB.

### 2.9 Preprocessing and statistical analysis

As we will use a mixed block/event-related design, all the following analyses (see Table 1 for a summary) will be conducted on the BOLD time-series at both the block and trial level: for the block-level analyses, we will contrast (1) task blocks (i.e., standard and deviant blocks combined) with silence blocks and (2) standard vs. deviant blocks. For the trial-level analysis, we will contrast (1) combined deviant and standard trials with BOLD extracted time-series of simulated time points during silence blocks and (2) deviant vs. standard trials. For the purpose of this project and the hypotheses presented, the analysis of RS data will not be discussed in this study.

**Table 1:** Hypotheses tested and corresponding analyses. This table provides a comprehensive overview of the analyses, brain areas, atlas employed, and statistical methods used for each hypothesis tested in this study.

Hypotheses tested	Analyses	Considered brain areas	Atlas	Statistical analysis
*Hypothesis 1*	Activation analysis (ROI-based)	All 7 sub-thalamic regions (L/R)	Oxford thalamic connectivity atlas	Small volume correction
*Hypothesis 2*	Activation analysis (ROI-based)	Primary auditory cortex (L/R): Heschl’s gyrus	Harvard-Oxford cortical and subcortical structural atlases	Small volume correction
*Hypothesis 3*	Activation analysis (ROI-based)	Secondary regions (L/R): superior temporal gyrus (both anterior and posterior division)	Harvard-Oxford cortical and subcortical structural atlases	Small volume correction
*Hypothesis 4*	ROI-to-ROI based connectivity	2 sub-thalamic (sensory and temporal) nuclei and Heschl’s gyrus	Oxford thalamic connectivity atlas/ Harvard-Oxford cortical and subcortical structural atlases	Subject-level: wGLM Group-level: LME
*Hypothesis 5*	ROI-to-ROI based connectivity	Heschl’s gyrus and superior temporal gyrus (both anterior and posterior division)	Harvard-Oxford cortical and subcortical structural atlases	Subject-level: wGLM Group-level: LME
*Hypothesis 6*	Voxel-to-voxel connectivity	Whole cortical and subcortical brain matters	No atlas	Subject-level: Intrinsic connective analysis Group-level: LME
*Hypothesis 7*	Spectrogram analysis	All 7 sub-thalamic regions (L/R); Primary auditory cortex (L/R): Heschl’s gyrus; Secondary regions (L/R): superior temporal gyrus (both anterior and posterior division)	Oxford thalamic connectivity atlas; Harvard-Oxford cortical and subcortical structural atlases; Harvard-Oxford cortical and subcortical structural atlases	Subject-level: Short Time Fourier Transform (STFT) for regional spectrogram estimation. Group-level: LME

#### 2.9.1 Preprocessing

fMRI data will be preprocessed in software SPM12 (Statistical Parametric Mapping, version 12, UCL Institute of Neurology, London, Britain, http://www.fil.ion.ucl.ac.uk/spm) and FSL 6.^3^ The preprocessing pipeline will include standard steps of realignment, susceptibility-induced distortions correction [FSL topup (Andersson et al., 2003; Smith et al., 2004)], slice acquisition time correction, coregistration, brain tissue segmentation, spatial normalization to the Montreal Neurological Institute stereotaxic template and smoothing using the Gaussian filter method with an isotropic kernel of size 6 mm. Outlier volumes, due to excessive head and body motion, will be detected using Artifact Detection Tools (ART) toolbox^4^ and will be regressed out in the first-level general linear model (GLM) analysis. An image will be defined as an outlier image or artifact if the absolute head displacement in the x-, y-, or z-direction is equal or greater than 2.3 mm, if the framewise displacement is greater than 0.4 mm, or if the overall average image intensity is greater than 3 standard deviations from the average intensity of the rest of the images. For each run of the 15-min auditory paradigm, the first five volumes will be discarded to allow magnetization to reach dynamic equilibrium.

#### 2.9.2 Activation analyses (ROI, Hypothesis 1-2-3)

First-level activation analysis will be performed using SPM 12 for block/trial levels. Activation values inside each ROI will be estimated using the Small Volume Correction (SVC) technique. The ROIs will include primary auditory cortex (Heschl’s gyrus (HG), including HG1 and HG2) and secondary auditory cortex (planum polare and planum temporale) extracted from the “Harvard-Oxford cortical and subcortical structural atlases”^5^, and Thalamic ROIs extracted from the “Oxford thalamic connectivity atlas,” in which sub-striatal regions are segmented according to their white-matter connectivity to cortical areas (Behrens T. et al. 2003; Behrens T. E. J. et al. 2003). We will include the areas of the thalamus labeled in the atlas as posterior-parietal, occipital, sensory, and prefrontal. First-level GLM design matrix will include six movement parameters and outlier volumes. Additionally, we will model the effect of elapsed time since awakening by adding a regressor based on the onsets of deviant and standard trials/blocks. The analysis will be considered significant at an alpha of <0.05, corrected for the number of ROIs.

#### 2.9.3 Connectivity analyses (ROI-ROI, Hypothesis 4-5)

Before carrying out the connectivity analysis, functional data will be denoised using a standard denoising pipeline (Nieto-Castanon, 2020) including the regression of potential confounding effects characterized by 5 principal components of white matter and cerebrospinal fluid using the component-based noise correction method (CompCor), 6 motions parameters and their 6 motions parameters and their first order derivatives, outliers volumes, session and task effects and their first order derivatives and linear trends within each functional run, followed by high pass filtering above 0.008 Hz. To assess task-related functional connectivity changes across experiment blocks/trials, the pair-wise ROI-ROI connectivity will be estimated as bivariate correlations using a weighted general linear model (wGLM) as implemented in CONN. The boxcar signal characterizing each block/trial, convolved with an SPM canonical hemodynamic response function and rectified, will be used to weight the ROI signals in the wGLM model. This will lead to block/trial-specific between-ROI correlation coefficients which will be then Fisher-transformed for further analysis. The ROIs will include two sub-thalamic (sensory and temporal) nuclei and the Heschl’s gyrus (hypothesis 4) and superior temporal gyrus (both anterior and posterior division; hypothesis 5). The results of this step would be connectivity matrices at the subject-level showing the correlation between the defined ROIs.

#### 2.9.4 Connectivity analyses (voxel-voxel, Hypothesis 6)

Data will be denoised as described in the previous paragraph. An exploratory analysis will be conducted at the whole-brain level, estimating the intrinsic connectivity maps related to each block/trial as implemented in CONN. This parameter characterizes network centrality at each voxel as the root mean square (RMS) of all short- and long-range connections between a voxel and the rest of the brain (Martuzzi et al., 2011).

#### 2.9.5 Group-level analysis

For group-level activation analysis (block/trial) we will perform a linear mixed effect model with nested random effects of subjects within the two experimental sessions, using package lme4 in software R. Fixed effects will be included to control for condition (CC vs. DC), stimulus/block type (deviant vs. standard or silence vs. sound), interaction between stimulus/block type and condition, propofol concentration and time to ROR. The latter allows the investigation of the effect of extending reports back in time, i.e., it is more probable that the subject was in a CC/DC state shortly before awakening as opposed to a long time before awakening. For the group-level connectivity analysis the same procedure will be followed, considering the connectivity matrices or intrinsic connectivity maps as dependent variables.

#### 2.9.6 fMRI spectral analysis

The progressive change in BOLD frequency content during the connected and disconnected consciousness conditions will be assessed using Short Time Fourier Transform. A sliding Hamming window will be used to calculate the spectrogram of each voxel’s time series (in order to check the validity of the results, we will repeat the analysis with variable window lengths of 50, 100, and 200 s corresponding to 60, 118, and 238 volumes, respectively). For each ROI, the BOLD power spectrogram will be calculated by averaging the power spectrogram across all voxels within the region. To ensure that different voxels within a brain region contributed equally to the power spectrogram of the region, the power spectrogram of individual voxels will be normalized by its total power. After estimating the ROI spectrograms, the peak frequency at each time point will be estimated based on the method introduced in Song et al. (2022). The time series showing the peak frequencies will be compared between the connected and disconnected consciousness conditions. This analysis will be performed in the ROIs used for hypotheses 1-2-3.

## 3 Data management and dissemination

Data will be stored in the NIfTI format, organized according to brain imaging data structure (BIDS) (Gorgolewski et al., 2016) and pseudo anonymized by an identification number, identifiable only by the researchers involved in the study. The results of the present study will be published in peer-reviewed journals as original research articles and will be presented at various scientific conferences. In these publications, the privacy of the individuals who took part will be safeguarded through anonymization.

## 4 Ethics

The study was approved by the University of Liege Hospital Ethics Committee (2020–707) and was registered at the European Union Drug Regulating Authorities Clinical Trials Database (identifier: 2020-003524-17). All study subjects will be informed in writing of the objectives, methods and potential risks of the experiment. They will be given two documents: a general information form on MRI acquisition and a specific form containing information on the study itself. All participants will provide written informed consent according to the Declaration of Helsinki and will receive financial compensation (300 euros). To ensure participant’s safety, vital parameters will be continuously monitored, and an anesthesiologist will be present in the MRI room for the entire duration of the experiment. Subjects will receive additional oxygen through a plastic facemask at a rate of 3 L.min^−1^. Monitored vital signs will include EKG and heart rate, non-invasive blood pressure, peripheral saturation in oxygen, inspired and expired CO_2_, thoracic movements amplitude, and respiratory rate. All material and medications needed to ensure safety of the sedation will be immediately available.

## 5 Discussion

Identifying the neuroimaging signatures of disconnected consciousness during sleep or anesthesia is a particularly difficult undertaking, owing to the inherent challenges in ascertaining the kind of experience (or lack thereof) a sleeping or sedated subject is having. This work aims to advance the investigation of the neural basis of sensory disconnection by achieving a more precise identification of this state, distinguishing it from states of unconsciousness and connected consciousness. In classical anesthesia studies, differentiation between these states was typically overlooked, with anesthetized subjects categorized as either conscious or unconscious based on behavioral responsiveness or explicit recall after anesthesia. In this study, we will overcome previous limitations by awakening participants immediately after auditory stimulation and asking them whether they were connected or disconnected before being awakened. This procedure, by minimizing amnesic effects of anesthesia and relying on subjective reports rather than (un)responsiveness to ascertain the subject’s state, enables a more accurate account of the subject’s experience.

This work represents also a notable progression in mitigating biases linked to contrastive, between-state paradigms, wherein two physiologically distinct states are compared (Koch et al., 2016; Boly et al., 2017). In this study, we ensure that participants are unresponsive and are in a dream-like state before awakening, in both connected and disconnected consciousness conditions. As a result, the conditions of connected and disconnected consciousness are contrasted within the same physiological state, i.e., both connected and disconnected participants are unresponsive and in a dream-like state.

Notwithstanding the surmounted challenges, certain methodological limitations within the present study design need to be addressed. As stated above, the differentiation between CC and DC is based on subjective reports. The limitations of introspection and thus of subjective reports to verify the state of consciousness have been widely discussed (Irvine, 2012; Tsuchiya et al., 2015). Objective measures of awareness, in which awareness is inferred from (above-chance) performance on a task, have often been advanced as more accurate and reliable measures for tracking changes in experience. However, it has been remarked that objective measures, instead of capturing subjective experience, only capture performance in the task, as below-chance performance does not necessarily imply that the subjects were unaware (Lau, 2008; Ellia et al., 2021). Which measure of awareness is best remains an open question at present. Regardless, our experimental design does not lend itself to the use of objective measures as participants are sedated and expected to remain unresponsive during the auditory stimulation, which makes task performance unfeasible. Furthermore, because the collection of subjective reports occurs during propofol sedation, the amnesic effects of anesthesia could lead participants to forget the experience they were having during the unresponsive period, therefore biasing the reports. Indeed, the absence of dream reports upon awakening does not necessarily imply unconsciousness or disconnectedness (Windt et al., 2016). At present, collecting retrospective reports is the only way to access participants’ subjective experience during unresponsive periods such as sleep or sedation. Amnesic effects on subjective reports are, however, significantly reduced the closer they are collected to the experience under investigation. Indeed, compared to post-anesthesia collection of reports, the serial awakening paradigm has been shown to minimize the lack of explicit recall as subjects are awakened and questioned about the experience they were having immediately before regaining responsiveness (Siclari et al., 2013, 2017). The extent to which reports can be extended back in time is however still unknown: e.g., if the participant reported being connected upon awakening, can we infer that (s)he was in a connected state during the entire 15 min of auditory stimulation or only during the last 5, 3 min or 60 s before awakening? This problem can however be partially accounted for by modelling the effect of time passing on the effects of interest. Finally, another limitation of our study pertains to its generalizability to alternative sensory modalities and different anesthetic agents. Our study explores sensory disconnection induced by the anesthetic agent propofol within the auditory modality. The extent to which our findings can be extended to other sensory modalities and anesthetics is presently unknown. Future studies incorporating a range of anesthetics and sensory modalities will therefore be necessary to validate and extend the applicability of these findings.

The findings of this study harbor the potential to disclose biomarkers of intraoperative connected consciousness, profoundly revolutionizing the landscape of anesthesia monitoring. A deeper understanding of the mechanisms that underlie states of disconnected consciousness will not only aid in devising strategies to induce it, as necessitated in instances such as surgical procedures, but also to suppress it, as demanded in scenarios like attention lapses, which pose as potential contributors to car accidents. Finally, these findings may be used to improve diagnosis of patients with disorders of consciousness. Understanding the level of consciousness and the cognitive capacities retained by these patients is, in fact, problematic due to their (often) limited responsiveness. Knowing the neural correlates of (dis)connectedness may allow to innovate the procedures of diagnosis and classification of these patients.

## Ethics statement

The study was approved by the University of Liege Hospital Ethics Committee (2020-707) and was registered at the European Union Drug Regulating Authorities Clinical Trials Database (identifier: 492 2020-003524-17). All participants will provide written informed consent according to the Declaration of Helsinki.

## Author contributions

BC: Conceptualization, Data curation, Formal analysis, Funding acquisition, Methodology, Software, Visualization, Writing – original draft. JM: Methodology, Writing – review & editing. SM: Formal analysis, Software, Writing – review & editing. RP: Formal analysis, Methodology, Writing – review & editing. RS: Methodology, Writing – review & editing. CP: Formal analysis, Methodology, Validation, Writing – review & editing. NA: Methodology, Writing – review & editing. ER: Writing – review & editing. AD: Writing – review & editing. MB: Conceptualization, Methodology, Writing – review & editing. MAB: Formal analysis, Writing – review & editing. LL: Methodology, Writing – review & editing. SL: Funding acquisition, Writing – review & editing. OG: Funding acquisition, Methodology, Writing – review & editing. VB: Funding acquisition, Methodology, Supervision, Writing – review & editing. JA: Conceptualization, Formal analysis, Funding acquisition, Methodology, Supervision, Writing – review & editing.

## Footnotes

^1^For brevity, the presence or lack of behavioral responses are written as (un)responsiveness.

^2^For brevity, to refer to both connected or disconnected consciousness we use (dis)connected consciousness.

^3^https://fsl.fmrib.ox.ac.uk/fsl/fslwiki/

^4^https://www.nitrc.org/projects/artifact_detect/

^5^https://identifiers.org/neurovault.collection:262
